# Work–Life Balance: Hopeless Endeavor or Rather, a True Privilege?

**DOI:** 10.3389/fped.2015.00099

**Published:** 2015-11-11

**Authors:** Tisha Wang

**Affiliations:** ^1^University of California Los Angeles, Los Angeles, CA, USA

**Keywords:** work–life balance, work–life conflict, women in science, women, working, intensivist

When I agreed to write this opinion piece on work–life balance, I quickly added it to my office whiteboard to-do list: “Work-Life Balance – October.” These four words generated much discussion – from jokes about the short timetable that I have to achieve the perfect work–life balance to conversations about how we are all trying to find work–life balance.

As a female academic intensivist and both an ICU director and program director, I am constantly surrounded by trainees and intensivists. Even before the whiteboard conversation piece, work–life balance was a frequent topic of conversation.

Point is – we all want work–life balance and think it is the key to our happiness, but the idea of it can somehow create more stress and anxiety. In fact, if you have thought too hard about it and whether or not you have it, you likely are feeling like a failure.

Like most physicians, I do not like feeling like a failure so instead I now think of it more like this: it is a privilege to have a fulfilling career, but that does not for a second lessen the importance of my personal life and the people that I care about.

Though work–life balance seems to be a goal for so many, most cannot even agree on the definition. Wikipedia defines it as proper prioritizing between “work” (career and ambition) and “lifestyle” (health, pleasure, leisure, family, and spiritual development/meditation).

When I ask my physician friends how they define work–life balance, to some it means having enough time to fulfill all of your work and home responsibilities and to be able to prioritize the important events in both domains. In short – be where you need to be and do what matters the most to you on a given day. For others, it means separating your work life from your home life so that neither really interferes with the other on a regular basis because they should indeed be separate entities. Yet another friend jokes that it means that work and life have equal importance and influence but speaking for herself, she has too much life and too much work so that the scale, while balanced, has actually broken.

All the definitions seem flawed and set us up for failure because there are often not enough hours in the day for all the people that want or need us, whether it is our family, friends, patients, or colleagues. As academic physicians, I see no way of advancing our careers and keeping work and life completely separate. And no one wants to feel that work and life are balanced but that they are being pushed to the limit. So, are we talking about a hopeless endeavor to even strive for work–life balance?

The work–life balance debate also undoubtedly becomes an emotional one because it is one of those topics that force us to put our core values into conflict. It makes us feel that we have to choose one core value (perhaps our dedication to our loved ones and even ourselves) over another (our ambition to make our mark in this world and advance our careers while helping others). Almost everyone, when asked, would say that nothing is more important to them than their family and friends. It is controversial to say or think otherwise but yet as academic physicians, there is little doubt that our careers are also incredibly important to us.

Work is often stimulating, inspiring, fulfilling, and where we spend much of our time. Many of us are doing it partially to support our families but regardless, work in academic medicine does give us purpose and a unique opportunity to impact many lives – through research, through training a generation of physicians, through being a caring physician to our patients. We do it because we believe in it and most of the time, I really do think of it as a privilege.

For me personally as a program director, I feel at any given time that I am just trying to raise 20 kids at work and guide them to be the best that they can be (full disclosure – I do not have children of my own). When classes graduate and leave every year, I mourn their departure, but seeing how they turn out is often worth the sacrifices I have had to make in my personal life. Not all aspects of my professional life are worth it however and I am constantly weighed down by the emotional turmoil that comes from having to choose between one important thing that is the “correct” important thing and another important thing that is still extremely important and offers a way to impact more lives.

Interestingly, during my occasional stints on social media, I see many articles about how millennials (I am a gen-Xer based on my age and work philosophy) in fact are no longer striving for work–life balance – #worklifebalance =oldnews (1). Instead, they are seeking a healthy work–life blend. Modern companies, such as Google, have created work environments full of gourmet meals and recreational areas. My own millennial trainees want a gym at work. Of course, the consequences of this millennial philosophy are largely unknown and it is plausible that the sense of being “on call” all the time and the constant multitasking could lead to further work–life imbalance (2).

For me personally, several things have been key to maintaining at least a sense of work–life balance: flexibility, the support and understanding of others, blending my work and life in a healthy way (as the millennials want to do), and strategic time management. Many women physicians with children choose academic medicine largely for the flexibility. They can leave during work hours for a school activity and then choose to work until 7 p.m. I personally opt to work at midnight instead of 7 a.m. when possible.

More controversial than that is that I choose to spend several hours per week while on vacation overseas to deal with the e-mail barrage that comes in while I am away. Some are quick to criticize that as a sign that I do not have work–life balance. But that is the flexibility I choose that helps create my own personal sense of balance and minimizes my work-related stress.

Next is the support and understanding of others – which arguably is a key component to every success in our lives, both at home and at work. As an intensivist, I can unexpectedly get stuck in the hospital because of a sick patient – similarly as an academic physician in leadership, I can also get stuck in a late meeting. If either of these events results in me missing dinner or something even more important than dinner, I, personally, am already bathing in stress and guilt. So in my personal life, I have made a concerted effort to surround myself with people that are largely supportive and understanding. No one needs to make me feel guiltier than I already feel. I consider myself lucky but I know that many physicians do not have this luxury.

Similarly in the hospital, I have worked hard to build a supportive faculty that largely cares about one another. Most of us are willing to help cover each other so we can all better fulfill our non-work priorities. As my colleague recently remarked, “We will all do better in the long run if we look out for one another.” As an intensivist, there is nothing worse than the feeling that you are rushing through a procedure or patient evaluation because you need to be elsewhere – several times in my career, I have had to make a concerted effort to slow down and relax so that the patient in front of me receives the care that they deserve. Having a supportive environment at work truly benefits everyone involved.

Another thing that sustains me is that I have found work–life blend after many years in academic medicine. Some of my closest friends are my colleagues – I can forget sometimes during a leisurely lunch that I am even working. Again this is a luxury but helps me immensely with feeling balanced at work, especially during a long or frustrating day.

Over the years, I have found that I truly do not mind integrating components of work into my life compartment and have stopped feeling guilty or judged for calling a patient after hours or texting my trainee back on a weekend that I am spending with my family. Really the one thing that we, as incredibly busy academic physicians, have to offer our loved ones and the people who depend on us at work is time. Balance is simply having the flexibility to choose how to divide your time without the constant feeling that someone is getting short-changed. Sometimes your life deserves more of your time – sometimes your work is what is taking up most of your time. Dividing your time in different ways at different points in your life is simply necessary and inevitable.

I personally try to allot my time to the people that will appreciate and benefit from it the most at any given time. I also block off time for my own sanity and rejuvenation so that I am better able to help others in my personal and professional life. I outsource tasks at home that I do not enjoy or have an aptitude for. I now choose projects at work more carefully. Is it a good use of my time that will lead to something meaningful for my career? Will the people that I give my time to appreciate it? Does it make more sense for me to delegate that task?

Whenever I think of achieving the perfect work–life balance, I am reminded of an article I read about Pepsi’s CEO, Indra Nooyi, who used strategies, such as having her secretaries monitor her children’s recreational privileges at home (3). She is often used as an example of a woman who has mastered work–life balance but has openly admitted that her own quest has been a struggle full of immense guilt. Perhaps half the battle is to acknowledge our limitations, cut ourselves some slack, and just do the best that we can. Our work colleagues, patients, and families/friends will hopefully understand.

As a last sentiment, I hope that academic medical centers realize the importance of providing an infrastructure of support that make our academic lives more sustainable. In an era of physician burnout and a high attrition of female faculty members at the associate professor level, I recently read with enthusiasm an article describing a new “time banking” program at Stanford’s Emergency Medicine Department in which performing traditionally uncompensated academic tasks, such as mentoring, teaching, and committee service, leads to “credits” that help maintain work–life balance in physicians’ lives by providing pre-arranged and compensated cleaning services, meals, childcare, and other household and work-related tasks (4–7). My institution provides childcare resources on campus and the option of “stopping the clock” in the context of academic promotion and childcare responsibilities. There should also be support and funding for physicians to attend conferences focused on physician wellness and work–life balance. I plan to attend the AAMC Mid-Career Women Faculty Professional Development Seminar this year and will definitely make it a priority in my career to promote innovative interventions to promote work–life balance that keep talented academic physicians where they belong.

For my fellow academic physicians – I hope that you are able to find a healthy work–life blend that involves flexibility, strategic time management, and a world at home and at work that is full of support, understanding, and meaning with less guilt (self-imposed and from others) when we are simply just doing the best that we can. In the end, we are the lucky ones who have found a sense of fulfillment in both our work and home lives. Our constant struggle to find the perfect work–life balance should thus be viewed as a privilege, as the struggle itself is simply a sign that we have a number of core values that hold great importance in our lives.

## Conflict of Interest Statement

The author declares that the research was conducted in the absence of any commercial or financial relationships that could be construed as a potential conflict of interest.

## References

[1] Loflin J Millenials Don’t Want Work-Life Balance. Linked In. (2015). Available from: www.linkedin.com/pulse

[2] Schawbel D Why Millennials Should Get Used to Work-Life Imbalance. Time. (2014). Available from: www.time.com

[3] McGregor J Indra Nooyi on Why Women can’t Have It All. The Washington Post (2014). Available from: www.washington.post.com

[4] Embriaco N Papazian L Kentish-Barnes N Pochard F Azoulay E. Burnout syndrome among critical care healthcare workers. Curr Opin Crit Care (2007) 13(5):482–8. 10.1097/MCC.0b013e3282efd28a 17762223

[5] Speck RM Sammel MD Troxel AB Cappola AR Williams-Smith CT Chittams J Factors impacting the departure rates of female and male junior medical school faculty: evidence from a longitudinal analysis. J Womens Health (2012) 21(10):1059–65. 10.1089/jwh.2011.3394 23004025

[6] Weinacker A Stapleton RD Still a man’s world, but why? Crit Care (2013) 17(1):113 10.1186/cc11915 23360580 PMC4055998

[7] Schulte B Time in the Bank: A Stanford Plan to Save Doctors from Burnout. The Washington Post (2015). Available from: www.washington.post.com

